# Evaluation of antimicrobial activities of plant aqueous extracts against *Salmonella* Typhimurium and their application to improve safety of pork meat

**DOI:** 10.1038/s41598-021-01251-0

**Published:** 2021-11-09

**Authors:** Alkmini Gavriil, Evangelia Zilelidou, Angelis-Evangelos Papadopoulos, Danae Siderakou, Konstantinos M. Kasiotis, Serkos A. Haroutounian, Chrysavgi Gardeli, Ilias Giannenas, Panagiotis N. Skandamis

**Affiliations:** 1 Laboratory of Food Quality Control and Hygiene, Department of Food Science and Human Nutrition, Agricultural University of Athens, Iera Odos 75, 11855 Athens, Greece; 2 Present Address: Laboratory of Pesticides’ Toxicology, Department of Pesticides Control and Phytopharmacy, Benaki Phytopathological Institute, 8 Stefanou Delta Street, Kifissia, 14561 Athens, Greece; 3 Laboratory of Nutritional Physiology and Feeding, Department of Animal Science, Agricultural University of Athens, Iera odos 75, 11855 Athens, Greece; 4 Laboratory of Food Chemistry and Analysis, Department of Food Science and Human Nutrition, Agricultural University of Athens, Iera Odos 75, 118 55 Athens, Greece; 5 Laboratory of Nutrition, School of Veterinary Medicine, Aristotle University of Thessaloniki, 54124 Thessaloniki, Greece

**Keywords:** Microbiology, Risk factors

## Abstract

Nine odorless laboratory-collected hydro-distilled aqueous extracts (basil, calendula, centrifuged oregano, corn silk, laurel, oregano, rosemary, spearmint, thyme) and one industrial steam-distilled oregano hydrolate acquired as by-products of essential oils purification were screened for their in vitro antimicrobial activity against three *Salmonella* Typhimurium strains (4/74, FS8, FS115) at 4 and 37 °C. Susceptibility to the extracts was mainly plant- and temperature-dependent, though strain dependent effects were also observed. Industrial oregano hydrolate eliminated strains immediately after inoculation, exhibiting the highest antimicrobial potential. Hydro-distilled extracts eliminated/reduced *Salmonella* levels during incubation at 4 °C. At 37 °C, oregano, centrifuged oregano, thyme, calendula and basil were bactericidal while spearmint, rosemary and corn silk bacteriostatic. A strain-dependent effect was observed for laurel. The individual or combined effect of marinades and edible coatings prepared of industrial hydrolate and hydro-distilled oregano extracts with or without oregano essential oil (OEO) was tested in pork meat at 4 °C inoculated with FS8 strain. Lower in situ activity was observed compared to in vitro assays. Marinades and edible coatings prepared of industrial oregano hydrolate + OEO were the most efficient in inhibiting pathogen. Marination in oregano extract and subsequent coating with either 50% oregano extract + OEO or water + OEO enhanced the performance of oregano extract. In conclusion, by-products of oregano essential oil purification may be promising alternative antimicrobials to pork meat stored under refrigeration when applied in the context of multiple hurdle approach.

## Introduction

Naturally derived antimicrobial compounds are increasingly gaining commercial attention as label-friendly alternatives to synthetic food preservatives^1^. Among them, phenolic compounds are a diverse group of plant secondary metabolites. They exhibit a wide range of physiological properties, including antimicrobial activity against a broad spectrum of pathogenic and spoilage bacteria^2,3^. Phenolic compounds are ubiquitous in plants^4^, with aromatic plants such as herbs and spices being especially rich in their phenolic content^5^. By-products of plant origin foods^4,6^ and essential oil industry^7^ are also good sources of phenolics. Given that the volume and economic burden of agro-industrial by-products processing are substantial^8^, their commercial exploitation as sources of phenolic compounds can provide an economical and environmentally-friendly way to enhance food safety^2,6^. Nevertheless, limited research is available regarding their potential antibacterial activity^2,6^.

The in vitro antimicrobial effect of plant extracts has been widely documented. However, fewer studies are available pertaining their in situ efficacy, probably due to the reduced effectiveness of plant extracts in food products^9^. Indeed, due to the complex and diverse nature of food environments, the extrapolation of the in vitro results to food products cannot be ensured^9^ at concentrations that maintain antimicrobial potential without compromising the sensory properties. Therefore, the in situ evaluation of their antimicrobial profile, as well as seeking alternative ways to optimize their efficacy, such as combining different treatments, are of utmost important for their systematic application in food matrices.

Phenolic compounds may either extend the shelf life of several food products^10,11^ or improve food safety, e.g., by promoting inactivation or growth inhibition of foodborne pathogens^7,12^. Among foodborne pathogens, *Salmonella* spp. is the second most common cause of reported zoonosis in Europe^13^ and the leading cause of hospitalization in the States^14^. With more than 2500 distinct serotypes, this pathogen consists a major health problem worldwide^15^, evolved to survive in a wide range of environments and across multiple hosts^16^. Its prevalence has been highly associated with products of animal origin, such as eggs, meat and poultry^13^. *S.* Typhimurium is one of the most common serovars isolated from pig meat^13^.

Differences in the innate characteristics among strains of the same species identically treated consist a major source of variation in microbiological studies, referred to as strain variability^17^. Differences in the phenotypic responses among strains of foodborne pathogens with regard to their inactivation potential can be extensive and therefore, should systematically be taken under consideration^18^.

Considering the above, the current study aimed primarily to screen the in vitro antimicrobial potential of nine laboratory (hydro-distilled) and one industrial (steam-distilled hydrolate) plant aqueous extracts acquired as by-products of essential oil purification procedure, against three strains of *Salmonella* spp. The second part aimed to evaluate the in situ antimicrobial activity of hydro-distilled oregano extract and industrial oregano hydrolate on improving the safety of pork meat against *Salmonella*.

## Results

### In vitro antimicrobial activity

Ten plant aqueous extracts acquired as by-products of essential oil production, either hydro-or steam-distilled, were screened for their in vitro antibacterial profiles against three strains of *S.* Typhimurium at 4 and 37 °C. The chemical composition of the extracts is presented in Table 1. Hydro-distilled aqueous extracts were consisted mainly of phenolic compounds, such as flavonoids and phenolic acids (e.g. hesperidin, luteolin, rosmarinic acid, chlorogenic acid). On the other hand, industrial oregano hydrolate collected by steam-distillation was composed of carvacrol (92.3%) and to a lesser extent of thymol (7.1%).

**Table 1:** Composition of hydro-and steam-distilled aqueous extracts based on UPLC-HESI-MS/MS and HPLC–DAD analyses, respectively.

Compound	Basil	Calendula	Centrifuged Oregano	Corn silk	Laurel	Oregano	Rosemary	Spearmint	Thyme	Industrial oregano
Adipic acid^a^	nd	32,250.0 ± 500.3	nd	nd	nd	nd	nd	nd	nd	nd
Apigenin^a^	nd	nd	nd	nd	nd	nd	nd	nd	nd	nd
Caffeic acid^a^	nd	nd		nd		nd	nd	nd	171.7 ± 20.5	nd
Catechin^a^	nd	nd	2449.1 ± 442.3	nd	**15,496.6 ± 2137.3**	nd	nd	nd	nd	nd
Chlorogenic acid^a^	38.9 ± 5.7	85.7 ± 10.2	74.1 ± 9.7	nd	nd	nd	30.3 ± 4.8	1604.0 ± 71.2	nd	nd
Diosmin^a^	nd		49.8 ± 12.6	nd	nd	nd	nd	795.0 ± 90.2	nd	nd
Ellagic acid^a^	nd	298.2 ± 17.5	nd	nd	nd	nd	nd	nd	nd	nd
Gallic acid^a^	nd	nd	nd	nd	479.0 ± 29.9	nd	nd	911.6 ± 34.9	nd	nd
Hesperidin^a^	nd	nd	nd	nd	nd	273.7 ± 37.6	nd	6576.1 ± 430.3	**2087.0 ± 315.6**	nd
Hyperoside^a^	nd	nd	nd	nd	nd	123.8 ± 20.8	nd	nd	nd	nd
Luteolin^a^	nd	**396.7 ± 31.0**	nd	393.4 ± 44.1	nd	nd	nd	404.6 ± 59.5	nd	nd
Myricetin^a^	nd	nd	nd	nd	nd	nd	87.1 ± 7.2	133.4 ± 15.0	nd	nd
Naringenin^a^	nd	nd	nd	nd	nd	27.5 ± 5.0	nd	nd	nd	nd
Orientin^a^	nd	nd	45.8 ± 8.0	579.4 ± 59.4	nd	nd	nd	4559.9 ± 235.5	nd	nd
Phloridzin^a^	nd	nd	nd	nd	nd	180.1 ± 17.2	nd	nd	nd	nd
Pinocembrin^a^	nd	nd	nd	nd	nd	nd	nd	34.7 ± 5.4	nd	nd
Protocatechuic acid^a^	nd	nd	**8759.5 ± 87.2**	nd	nd	nd	nd	nd	nd	nd
Quercetin^a^	nd	nd	nd	233.2 ± 39.2	nd	nd	nd	nd	nd	nd
Rosmarinic acid^a^	**12,164.7 ± 435.6**	nd	1610.6 ± 90.8	**3451.3 ± 120.3**	nd	**2730.3 ± 195.5**	**1783.6 ± 170.0**	**54,977.7 ± 2301.8**	212.4 ± 40.4	nd
Rutin^a^	nd	nd	nd	nd	1961.2 ± 203.8	382.0 ± 41.4	nd	1961.2 ± 69.9	nd	nd
Syringic acid^a^	nd	nd	344.6 ± 46.8	nd	nd	nd	nd	31,994.2 ± 1002.6	nd	nd
Carvacrol^b^	nd	nd	nd	nd	nd	nd	nd	nd	nd	92.3
Thymol^b^	nd	nd	nd	nd	nd	nd	nd	nd	nd	7.1

The main compound of each extract is indicated in bold.

nd: not detected.

^a^ng/g.

^b^%.

**Figure 1:**
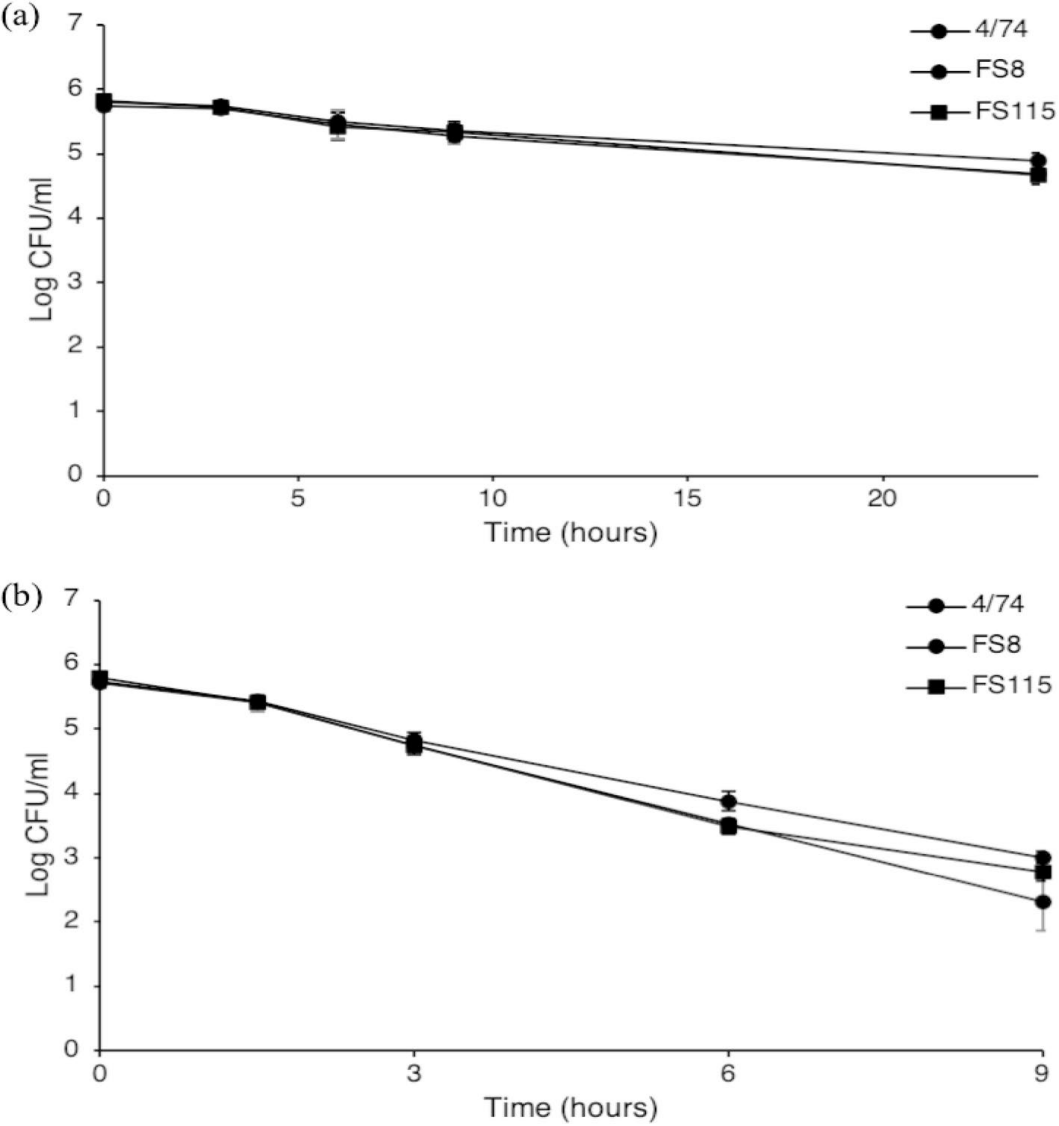
In vitro inactivation of *S.* Typhimurium 4/74, FS8 and FS115 in TSB adjusted to pH 3.5 with HCl 6 N at 4 °C (**a**) and 37 °C (**b**). Each data point is a mean of 6 replicates (± standard deviation).

**Figure 2:**
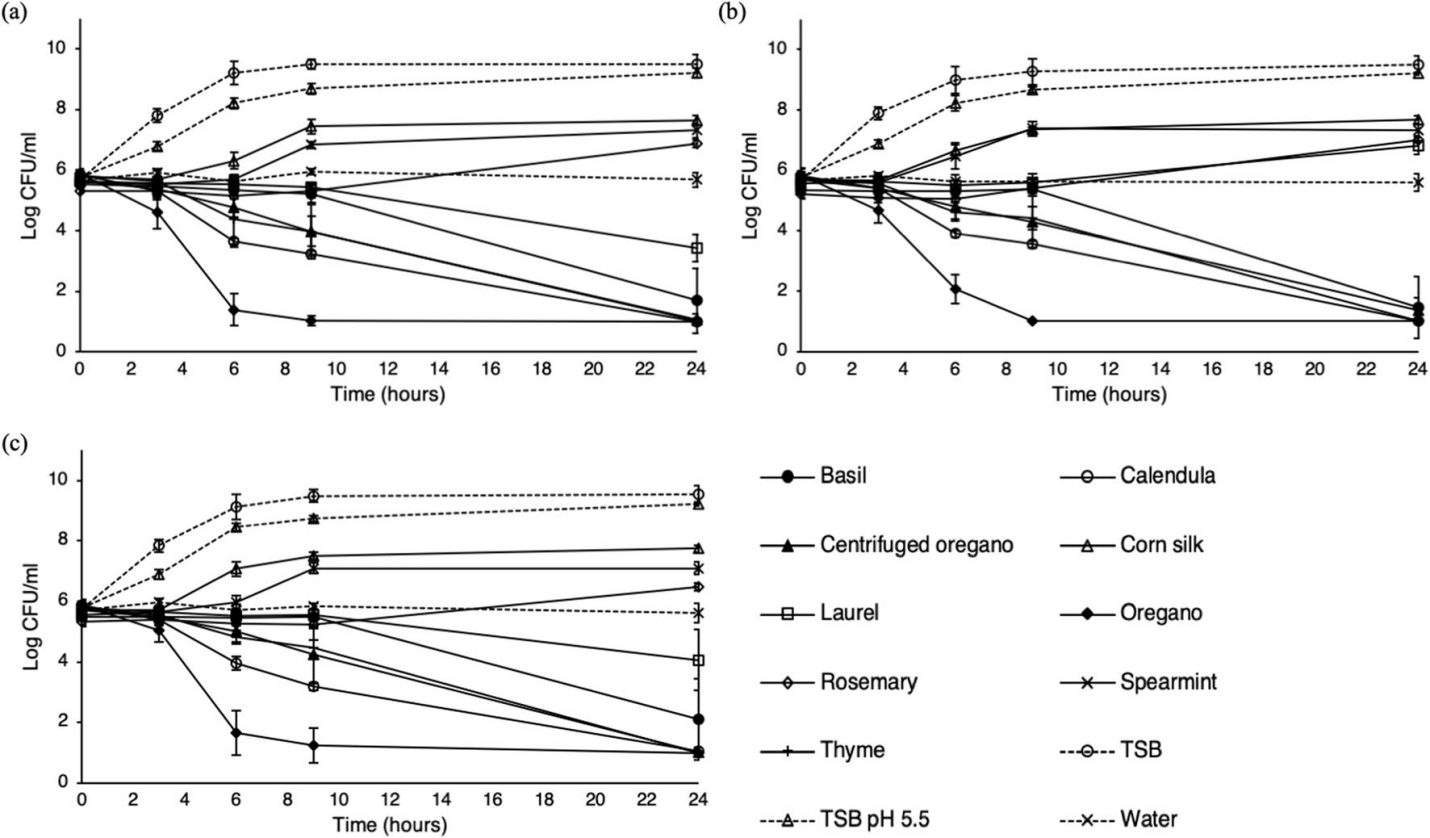
In vitro inactivation or growth inhibition kinetics of *S.* Typhimurium (**a**) 4/74, (**b**) FS8 and (**c**) FS115 in nine different plant aqueous extracts incubated at 37 °C. Extraction was carried out by hydro-distillation. TSB, TSB adjusted to pH 5.5 and de-ionized water were used as controls. Each data point is a mean of at least 6 replicates (± standard deviation).

A marked variability was observed in the antimicrobial potential of the extracts. Their antimicrobial activity was mainly dependent on plant and incubation temperature. Industrial oregano hydrolate had the highest bactericidal activity, irrespectively of the incubation temperature. It reduced strains cell densities below the threshold of detection (1.3 log CFU/ml) immediately after inoculation. On the other hand, enumeration of cells inoculated in TSB adjusted to pH 3.5 was performed for several hours after incubation at both 4 and 37 °C (Fig. 1), indicating a higher antimicrobial potential of industrial oregano hydrolate compared to pH controls. Among the hydro-distilled aqueous extracts, oregano exhibited the strongest antimicrobial activity in both temperatures, followed by thyme, calendula and centrifuged oregano extracts with activities that varied dependent on the temperature and the strain used (Table 2 and Fig. 2). On the other hand, corn silk had the lowest impact on the survival of *Salmonella* strains within the incubation period at both temperatures (Table 2 and Fig. 2). At 4 °C, all extracts effectively reduced pathogen levels. The time needed for a four-log reduction (t_4D_) is presented at Table 2, whereas the order of antimicrobial potential based on the calculated t_4D_ estimates is given in supplementary data (see supplementary Table S3 online). Among the three strains, small differences (*P* < 0.05) in their sensitivity were observed when exposed to basil, calendula, c. oregano and corn silk (Table 2). Cultures inoculated in TSB and TSB pH 5.5 remained practically stable to their initial levels, with populations ranging between 5.0 and 5.8 logs throughout incubation period (Table 3). On the other hand, an approximately 2.0 log reduction was observed during incubation to de-ionized water (Table 3), although this reduction was lower compared to the effect of the extracts (Table 2).

**Table 2:** In vitro inactivation kinetics (t_4D_ estimates) of *S.* Typhimurium 4/74, FS8 and FS115 in nine different plant aqueous extracts incubated at 4 °C.

Plant extract	Strain								
	4/74			FS8			FS115		
	t_4D_	RMSE	R^2^	t_4D_	RMSE	R^2^	t_4D_	RMSE	R^2^
Basil	16.23 ± 076 **(B, f)**	0.1286 ± 0.0216	0.9860 ± 0.055	16.95 ± 0.90 **(B, f)**	0.1901 ± 0.0405	0.9688 ± 0.0117	18.69 ± 1.43 **(A, e)**	0.1166 ± 0.0361	0.9863 ± 0.0073
Calendula	8.22 ± 0.21 **(B, c)**	0.3102 ± 0.0483	0.9727 ± 0.079	9.29 ± 0.26 **(A, c)**	0.1929 ± 0.0567	0.9858 ± 0.0091	7.91 ± 0.23 **(B, b)**	0.3424 ± 0.0714	0.9708 ± 0.0121
C. Oregano	11.86 ± 0.89 **(B, d)**	0.2429 ± 0.0557	0.9834 ± 0.0063	12.48 ± 0.38 **(B, d)**	0.2468 ± 0.0575	0.9811 ± 0.0080	13.90 ± 0.18 **(A, c)**	0.2025 ± 0.0521	0.9847 ± 0.0080
Corn silk	39.38 ± 1.47 **(B, h)**	0.2421 ± 0.0590	0.9793 ± 0.076	41.58 ± 1.07 **(A, h)**	0.2284 ± 0.0305	0.9794 ± 0.0063	43.39 ± 1.71 **(A, g)**	0.2025 ± 0.0347	0.9832 ± 0.0059
Laurel	21.27 ± 1.37 **(A, g)**	0.1481 ± 0.0429	0.9936 ± 0.0035	24.12 ± 2.68 **(A, g)**	0.2780 ± 0.0287	0.9750 ± 0.0070	22.69 ± 2.01 **(A, f)**	0.2396 ± 0.1165	0.9811 ± 0.0175
Oregano	3.09 ± 0.62 **(A, a)**	0.3468 ± 0.0618	0.9806 ± 0.0081	3.24 ± 0.32 **(A, a)**	0.3722 ± 0.1488	0.9729 ± 0.0154	3.06 ± 0.42 **(A, a)**	0.2695 ± 0.1527	0.9849 ± 0.0138
Rosemary	14.54 ± 0.90 **(A, e)**	0.3367 ± 0.0677	0.9671 ± 0.0123	16.56 ± 1.14 **(A, ef)**	0.3136 ± 0.0414	0.9677 ± 0.0057	16.53 ± 0.87 **(A, d)**	0.2279 ± 0.0617	0.9803 ± 0.0102
Spearmint	14.56 ± 0.88 **(A, e)**	0.2726 ± 0.1579	0.9748 ± 0.207	14.83 ± 0.41 **(A, e)**	0.2369 ± 0.1351	0.9789 ± 0.200	15.01 ± 0.24 **(A, cd)**	0.2035 ± 0.0774	0.9826 ± 0.0125
Thyme	6.26 ± 0.79 **(A, b)**	0.2725 ± 0.1493	0.9837 ± 0.0189	6.15 ± 1.04 **(A, b)**	0.1829 ± 0.304	0.9936 ± 0.0018	6.13 ± 1.06 **(A, b)**	0.1201 ± 0.0642	0.9971 ± 0.0028

Extraction was carried out by hydro-distillation. t_4D_’s were calculated by fitting the log-transformed data to the Weibull model. Each value is a mean of at least 6 replicates (± standard deviation). Goodness of the fitting was evaluated using regression coefficient (R^2^) and root-mean square error (RMSE).

Different lowercase letters within the same column indicate statistical differences (*P* < 0.05) for a single strain inoculated in different plant extracts according to Tukey’s HSD.

Different capital letters within each row indicate statistical differences (*P* < 0.05) among different strains inoculated to the same plant extract according to Tukey’s HSD.

**Table 3:** In vitro effect of controls (TSB, TSB adjusted to pH 5.5 and de-ionized water) in *S.* Typhimurium 4/74, FS8 and FS115 incubated at 4 °C.

Strain		Controls		
	Time (days)	TSB	TSB pH 5.5	Water
4/74	0	5.8 ± 0.3 **(a)**	5.8 ± 0.1 **(a)**	5.8 ± 0.2 **(a)**
	8	5.8 ± 0.2 **(a)**	5.8 ± 0.1 **(a)**	5.6 ± 0.2 **(a)**
	16	5.7 ± 0.1 **(a)**	5.7 ± 0.1 **(a)**	5.6 ± 0.2 **(a)**
	21	5.4 ± 0.2 **(ab)**	5.8 ± 0.1 **(a)**	4.6 ± 0.3 **(b)**
	27	5.4 ± 0.2 **(ab)**	np	4.4 ± 0.5 **(bc)**
	32	5.4 ± 0.2 **(ab)**	5.7 ± 0.1 **(a)**	4.6 ± 0.1 **(b)**
	38	5.3 ± 0.0 **(b)**	5.7 ± 0.1 **(ab)**	4.1 ± 0.3 **(cd)**
	45	5.3 ± 0.1 **(b)**	5.6 ± 0.1 **(b)**	3.9 ± 0.3 **(d)**
FS8	0	5.8 ± 0.3 **(a)**	5.8 ± 0.1 **(ab)**	5.8 ± 0.2 **(a)**
	8	5.8 ± 0.2 **(a)**	5.8 ± 0.1 **(ab)**	5.7 ± 0.2 **(a)**
	16	5.6 ± 0.2 **(ab)**	5.8 ± 0.2 b **(a)**	5.7 ± 0.2 **(a)**
	21	5.3 ± 0.2 **(bc)**	5.8 ± 0.1 **(abc)**	4.9 ± 0.4 **(b)**
	27	5.4 ± 0.1 **(bc)**	np	4.6 ± 0.4 **(b)**
	32	5.4 ± 0.1 **(abc)**	5.7 ± 0.1 **(abc)**	4.5 ± 0.5 **(b)**
	38	5.5 ± 0.2 **(abc)**	5.6 ± 0.1 **(bc)**	3.9 ± 0.1 **(c)**
	45	5.1 ± 0.0 **(c)**	5.6 ± 0.1 **(c)**	3.6 ± 0.3 **(c)**
FS115	0	5.9 ± 0.3 **(a)**	5.7 ± 0,1 **(ab)**	5.8 ± 0.2 **(a)**
	8	5.7 ± 0.1 **(ab)**	5.8 ± 0.1 **(a)**	5.6 ± 0.2 **(a)**
	16	5.4 ± 0.2 **(abc)**	5.7 ± 0.2 **(abc)**	5.2 ± 0.3 **(b)**
	21	5.4 ± 0.2 **(abc)**	5.7 ± 0.1 **(abc)**	4.8 ± 0.3 **(c)**
	27	5.3 ± 0.1 **(bc)**	np	4.6 ± 0.4 **(c)**
	32	5.2 ± 0.0 **(c)**	5.6 ± 0.1 **(abc)**	3.8 ± 0.3 **(d)**
	38	5.2 ± 0.1 **(bc)**	5.5 ± 0.2 **(bc)**	3.6 ± 0.3 **(d)**
	45	5.0 ± 0.4 **(c)**	5.5 ± 0.1 **(c)**	3.8 ± 0.1 **(d)**

Each data point is a mean of at least 6 replicates (± standard deviation).

Different lowercase letters within each column of the same strain indicate statistical differences (*P* < 0.05) during incubation at 4 °C according to Tukey’s HSD.

np: not performed.

At 37 °C, oregano, centrifuged oregano, thyme, calendula and basil decreased pathogen population within 24 h of incubation (Fig. 2). Oregano almost eliminated initial populations within the first 9 h of incubation. Basil, calendula, centrifuged oregano and thyme extracts, also reduced/inactivated *Salmonella* within 24 h of incubation, though at a lower rate compared to oregano (Fig. 2). On the other hand, corn silk, spearmint and rosemary had only a bacteriostatic effect, permitting pathogen growth in a lower growth rate and up to lower final populations (*P* < 0.05) compared to controls (TSB and TSB adjusted to pH 5.5) (Fig. 2). At the end of incubation period (24 h), the highest number of survivors were found in corn silk, whereas lower log counts (*P* < 0.05) were enumerated in rosemary (Fig. 2, see Supplementary Table S2 online). A strain-dependent effect was observed for laurel aqueous extract: FS8 strain was increased by 1.2 logs, while strains 4/74 and FS115 were reduced by 2.3 and 1.5 logs, respectively (Fig. 2, see Supplementary Table S2 online). Populations among the three strains inoculated to the same plant extract (apart from laurel) and for the same time interval, though significant in some cases, did not exceed 0.5–0.8 logs (Fig. 2, see Supplementary Table S2 online).

Overall, the aqueous extracts effectively reduced the in vitro levels or growth rate of three *S.* Typhimurium strains, in a plant-, temperature- and strain-dependent manner.

### Antimicrobial activity of hydro-distilled oregano extract and industrial oregano hydrolate against *S.* Typhimurium FS8 in pork meat

Hydro-distilled oregano extract and industrial oregano hydrolate were further examined for their efficacy in improving the safety of pork meat stored at 4 °C. These extracts were selected since they exhibited the strongest in vitro antimicrobial performance. The individual or combined effect of marination and edible coatings supplemented with or without low concentrations of OEO was assessed for the application of the extracts on the meat surface. Since the in vitro inactivation profiles of the three strains did not significantly differ during their exposure to oregano at 4 °C, FS8 strain (minced pork isolate) was chosen to be inoculated to the pork meat samples (in situ exposure to the oregano extracts).

### Effect of marination

**Figure 3:**
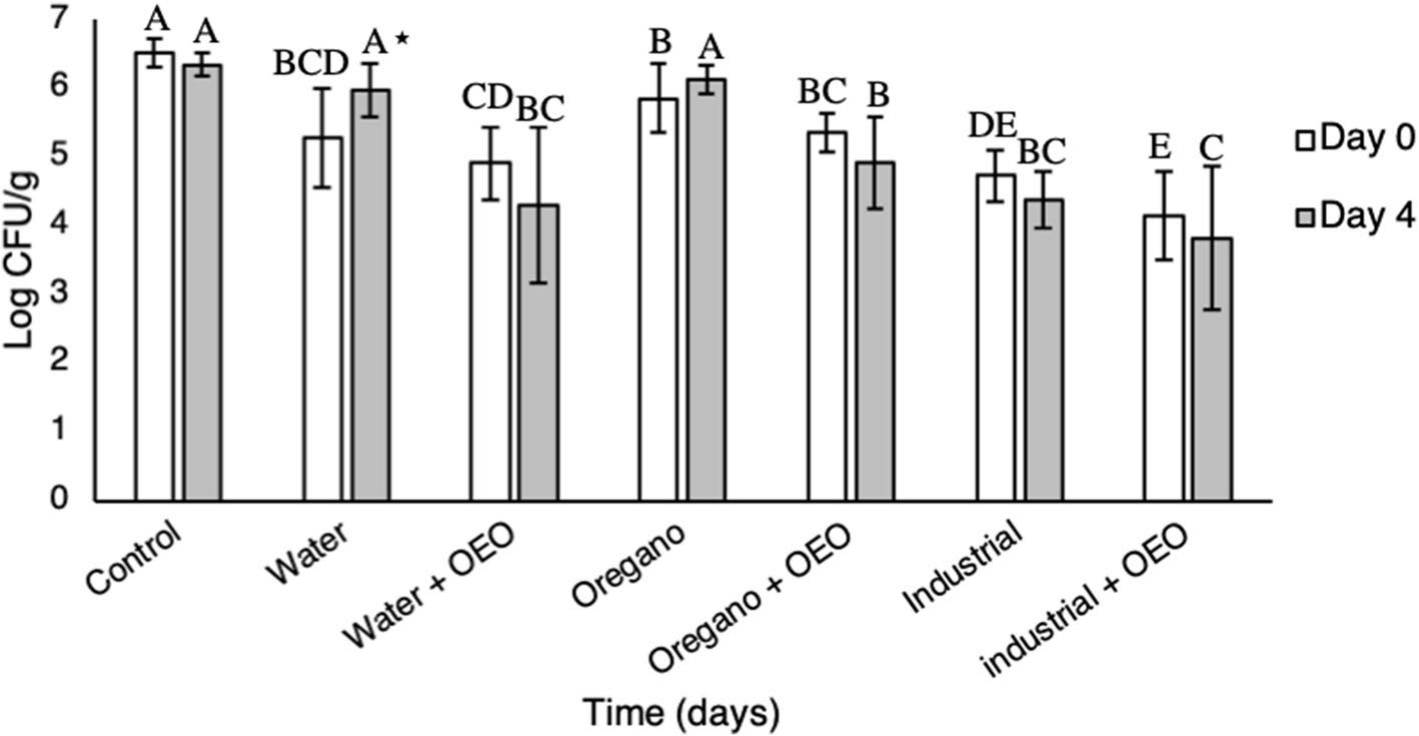
Effect of 3-h marination on the survival of *S.* Typhimurium FS8 on pork meat. Hydro-distilled oregano extract and industrial oregano hydrolate supplemented with or without 0.2% oregano essential oil (OEO) were used as marination solutions. Water and inoculated untreated pork meat samples were used as controls. Samples were stored at 4 °C. Sampling was performed immediately after treatment (white bars) and after 4 days of storage (grey bars). Bars represent an average (± standard deviation) of six replicates. Different capital letters indicate statistical differences (*P* < 0.05) among different treatments of the same storage time according to Tukey’s HSD. Star indicate statistical differences (*P* < 0.05) between identically treated samples during 0 and 4th day of storage according to *t-test*.

Depending on the treatment, application of 3 h marination resulted in 0.6–2.4 log reductions (*P* < 0.05) of the initial bacterial population (Fig. 3). When samples were marinated in water and water + OEO, a 1.2 and 1.6 log reduction (*P* < 0.05), respectively, was observed compared to the inoculated untreated samples immediately after the application of the treatment. No further reduction (*P* < 0.05) of *Salmonella* populations was observed when oregano, oregano + OEO or industrial oregano hydrolate were applied as marination solutions. On the contrary, industrial oregano hydrolate + OEO had the highest antimicrobial activity (*P* < 0.05), lowering the initial bacterial population by 2.4 log CFU/g compared to the inoculated untreated samples and by ~ 1 log CFU/g compared to the samples marinated in water + OEO (Fig. 3, see Supplementary Table S4 online). Significant differences were not observed between samples marinated in extracts with and those without OEO (*P* > 0.05). Furthermore, there was not any residual antimicrobial activity after storage at 4 °C for 4 days, regardless of the marination solution (*P* > 0.05) (Fig. 3, see Supplementary Table S4 online). Therefore, marination of pork meat samples resulted in a rapid decrease of *Salmonella* populations, especially when industrial + OEO was used in the marinades.

### Effect of edible coatings

**Figure 4:**
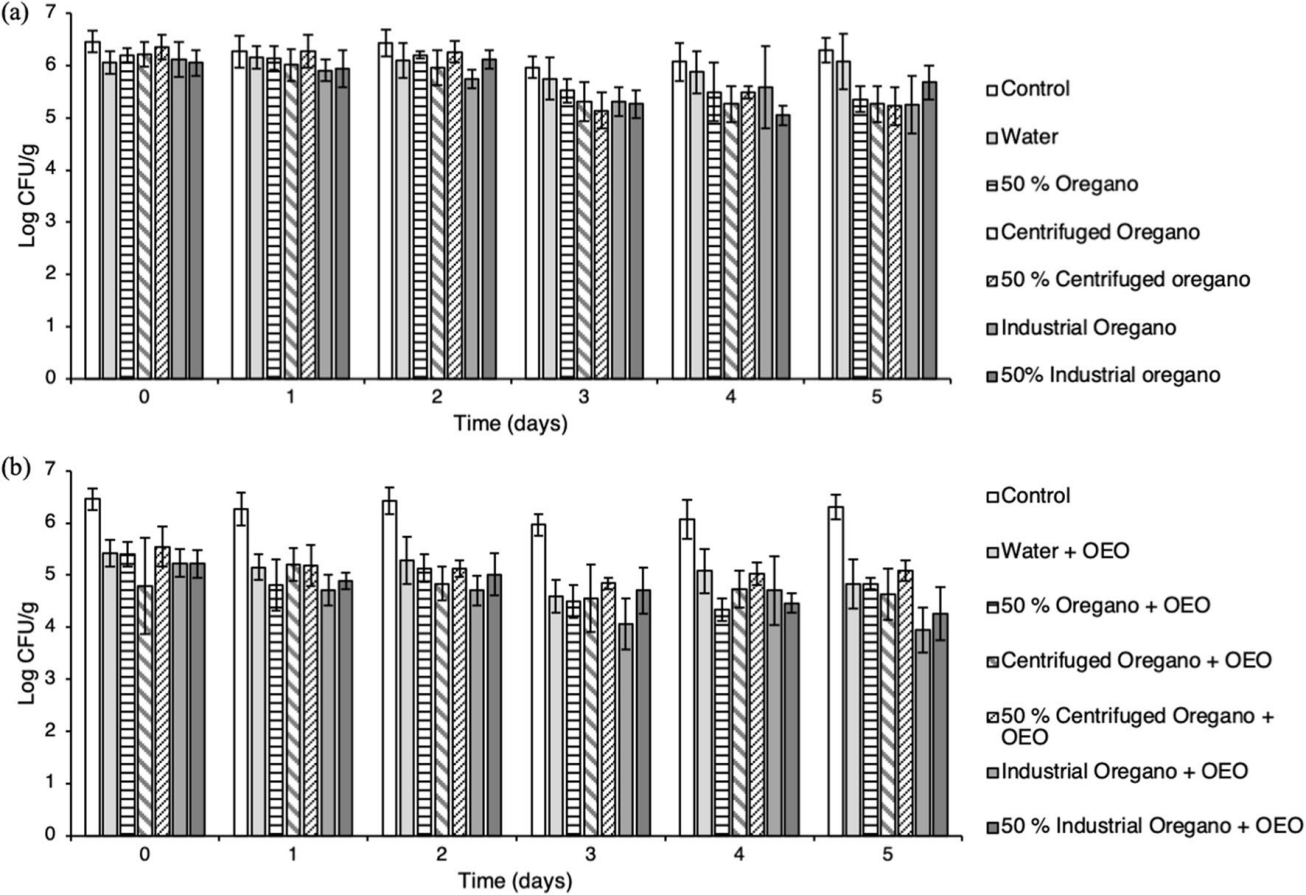
Effect of edible coatings prepared of water, hydro-distilled oregano extract and industrial oregano hydrolate supplemented without (**a**) or with (**b**) 0.5% oregano essential oil (OEO) on the survival of *S.* Typhimurium FS8 inoculated in pork meat at 4 °C. Inoculated uncoated pork meat samples were used as controls. Sampling was performed immediately after treatment and up to 5 days of storage. Each bar represents an average (± standard deviation) of six replicates.

The effect of sodium alginate edible coatings prepared of water, 50% oregano, centrifuged oregano, 50% centrifuged oregano, industrial oregano and 50% industrial oregano supplemented with or without 0.5% OEO on pathogen survival was studied (Fig. 4). Application of coatings with no added OEO resulted in ~ 1.0 log CFU/g reduction (*P* < 0.05) during storage for all extract-treated samples (Fig. 4a, see Supplementary Table S5 online). At the end of storage period, no differences (*P* > 0.05) were found among the extract-coated samples, though lower survivors were enumerated compared to uncoated and water-coated meat samples (*P* < 0.05) (Fig. 4a, see Supplementary Table S5 online).

Supplementation of edible coatings with OEO was initially performed using a concentration of 0.2% (data not shown). Since no effect was found, a higher concentration of 0.5% was tested. Incorporation of 0.5% OEO increased the antibacterial effect of the coatings compared to those with no added OEO: all treated samples had 1.0–1.5 log (*P* < 0.05) lower bacterial levels compared to the inoculated untreated samples immediately after treatment, regardless of the film-forming solution (Fig. 4b, see Supplementary Table S6 online). In addition, a gradual reduction of up to 1.3 log CFU/g (*P* < 0.05) was observed at the end of storage period (5th day of storage) for most of the samples tested, apart from those coated in centrifuged oregano + OEO (*P* > 0.05). Nonetheless, coatings prepared of industrial oregano hydrolate + OEO were the most potent in decreasing the levels of *Salmonella*, resulting in a total reduction of 2.6 log CFU/g on day 5 compared to inoculated uncoated samples and ~ 1 log CFU/g compared to samples coated with water + OEO (Fig. 4b, see Supplementary Table S6 online). Therefore, addition of low levels of OEO enhanced the antimicrobial activity of industrial oregano hydrolate incorporated through edible coatings.

### Combined effect of marination and edible coatings

The combined effect of antimicrobial marination and edible coatings on the survival of *S.* Typhimurium FS8 on pork meat was evaluated (Table 4). Edible coatings enhanced the antimicrobial effect of marination for some of the treatments tested, resulting in log reductions during storage. Combination of marination in hydro-distilled oregano extract and water + OEO coatings or hydro-distilled 50% oregano + OEO coatings enhanced the performance of the extract, leading to a reduction of 0.9 and 1.4 log units (*P* < 0.05) during storage, respectively. The total reduction was 1.4 and 2.0 log CFU/g, respectively, compared to the inoculated untreated pork meat samples (Table 4).

**Table 4:** Combined effect of marinades and edible coatings prepared of water, hydro-distilled oregano extract and industrial oregano hydrolate supplemented with or without 0.2% OEO in the inactivation of *S.* Typhimurium FS8 in pork meat at 4 °C.

Marination	Coatings	Days		
		0	2	4
No	No	6.5 ± 0.2 **a**	6.2 ± 0.6 **a**	6.3 ± 0.2 **a**
Water	Water	5.3 ± 0.7 **A, b**	5.5 ± 0.2 **A, abc**	5.1 ± 0.5 **A, bc**
	Water + OEO	5.3 ± 0.7 **A, b**	4.5 ± 0.3 **A, d**	4.9 ± 0.5 **A, c**
	50% Oregano	5.3 ± 0.7 **A, b**	5.5 ± 0.4 **A, ab**	5.8 ± 0.4 **A, ab**
	**50% Oregano + OEO**	**5.3 ± 0.7 A, b**	**4.9 ± 0.8 AB, bcd**	**4.4 ± 0.4 B, c**
	Industrial	5.3 ± 0.7 **A, b**	4.6 ± 0.3 **A, cd**	4.9 ± 9.5 **A, c**
	Industrial + OEO	5.3 ± 0.7 **A, b**	4.6 ± 0.5 **A, d**	5.0 ± 0.6 **A, c**
Water + OEO	**Water**	**4.9 ± 0.6 A, b**	**4.3 ± 0.2 B, b**	**4.2 ± 0.6 B, bc**
	**Water + OEO**	**4.9 ± 0.6 A, b**	**4.0 ± 0.3 B, b**	**3.7 ± 0.5 B, c**
	**50% Oregano**	**4.9 ± 0.6 A, b**	**4.2 ± 0.1 B, b**	**4.7 ± 0.3 AB, b**
	**50% Oregano + OEO**	**4.9 ± 0.6 A, b**	**3.7 ± 1.0 B, b**	**3.9 ± 0.3 B, c**
	**Industrial**	**4.9 ± 0.6 A, b**	**4.2 ± 0.7 AB, b**	**3.9 ± 0.3 B, c**
	**Industrial + OEO**	**4.9 ± 0.6 A, b**	**3.5 ± 1.0 B, b**	**4.1 ± 0.5 AB, bc**
Oregano	Water	5.9 ± 0.5 **A, b**	6.1 ± 0.8 **A, a**	5.5 ± 0.7 **A, ab**
	**Water + OEO**	**5.9 ± 0.5 A, b**	**5.5 ± 0.3 AB, a**	**5.1 ± 0.6 B, bc**
	50% Oregano	5.9 ± 0.5 **A, b**	5.7 ± 0.6 **A, a**	5.8 ± 0.2 **A, ab**
	**50% Oregano + OEO**	**5.9 ± 0.5 A, b**	**5.5 ± 0.7 A, a**	**4.5 ± 1.0 B, c**
	Industrial	5.9 ± 0.5 **A, b**	5.5 ± 0.4 **A, a**	5.4 ± 0.3 **A, abc**
	Industrial + OEO	5.9 ± 0.5 **A, b**	5.2 ± 0.5 **A, a**	5.2 ± 0.5 **A, bc**
Oregano + OEO	Water	5.4 ± 0.3 **A, b**	5.4 ± 0.7 **A, ab**	4.8 ± 0.4 **A, b**
	**Water + OEO**	**5.4 ± 0.3 A, b**	**4.8 ± 0.5 B, bc**	**4.7 ± 0.7 B, bc**
	**50% Oregano**	**5.4 ± 0.3 A, b**	**4.0 ± 0.7 B, c**	**4.5 ± 0.8 B, bc**
	**50% Oregano + OEO**	**5.4 ± 0.3 A, b**	**4.3 ± 1.0 B, bc**	**4.0 ± 0.6 B, c**
	**Industrial**	**5.4 ± 0.3 A, b**	**4.6 ± 0.2 B, bc**	**4.4 ± 0.1 B, bc**
	**Industrial + OEO**	**5.4 ± 0.3 A, b**	**4.8 ± 0.5 B, bc**	**4.4 ± 0.4 B, bc**
Industrial	Water	4.7 ± 0.4 **A, b**	4.1 ± 0.8 **A, b**	3.8 ± 0.9 **A, b**
	**Water + OEO**	**4.7 ± 0.4 A, b**	**4.0 ± 0.3 AB, b**	**3.8 ± 1.0 B, b**
	50% Oregano	4.7 ± 0.4 **A, b**	4.4 ± 0.4 **A, b**	4.4 ± 0.4 **A, b**
	50% Oregano + OEO	4.7 ± 0.4 **A, b**	4.4 ± 0.2 **A, b**	4.4 ± 0.3 **A, b**
	Industrial	4.7 ± 0.4 **A, b**	4.5 ± 0.2 **A, b**	4.4 ± 0.2 **A, b**
	Industrial + OEO	4.7 ± 0.4 **A, b**	4.1 ± 0.8 **AB, b**	3.9 ± 0.3 **B, b**
Industrial + OEO	Water	4.1 ± 0.6 **A, b**	4.2 ± 0.4 **A, b**	4.0 ± 0.8 **A, b**
	Water + OEO	4.1 ± 0.6 **A, b**	4.1 ± 0.4 **A, b**	3.8 ± 0.7 **A, b**
	50% Oregano	4.1 ± 0.6 **A, b**	4.4 ± 0.7 **A, b**	4.5 ± 0.4 **A, b**
	**50% Oregano + OEO**	**4.1 ± 0.6 AB, b**	**4.4 ± 0.2 A, b**	**3.3 ± 0.7 B, b**
	Industrial	4.1 ± 0.6 **A, b**	3.9 ± 0.6 **A, b**	4.4 ± 0.5 **A, b**
	Industrial + OEO	4.1 ± 0.6 **A, b**	3.9 ± 0.3 **A, b**	4.2 ± 0.4 **A, b**

Antimicrobial combinations leading in reductions during storage are indicated in bold. Each value represents an average (± standard deviation) of six replicates.

Different capital letters within the same row indicate statistical differences (*P* < 0.05) of samples during storage at 4 °C according to Tukey’s HSD.

Different lowercase letters within the same column indicate statistical differences (*P* < 0.05) among samples marinated in the same solution and covered with different coatings according to Tukey’s HSD.

In all experiments, no *Salmonella* populations were detected in none of the uninoculated untreated meat samples.

### Changes in the pH during storage

Slight/no alterations were recorded at the pH values of most treated samples. On the contrary, pH of untreated samples increased from 5.8 to approximately 6.4–6.5 during storage (see Supplementary Figs. S1–S3 online).

## Discussion

The last decades, natural antimicrobials, such as essential oils and plant extracts other than essential oils, are increasingly gaining scientific and commercial attention as label-friendly alternatives to synthetic food preservatives. So far, the majority of the scientific research regarding the effect of natural antimicrobials has been focused on essential oils^19–22^. Despite the perceived inhibitory effect of some of these naturally occurring substances, their practical application is often restricted due to their strong flavor and aroma, their application cost and their potential toxicity in human health when applied in high levels^23^. On the other hand, the bactericidal activity of plant derived extracts other than essential oils has been attributed to the presence of polyphenolic fraction^5,7,24^, which has a reported antimicrobial effect against foodborne bacteria, e.g. *Salmonella* spp., *Listeria monocytogenes, Staphylococcus aureus* and *Escherichia coli*^25–31^. Polyphenols can be obtained from agricultural by-products and food waste and used for several applications due to their antioxidant and antimicrobial activities^32^. Among the potential sources, by-products of essential oil distillation are rich in phenolic compounds and have been found effective in delivering an important bactericidal effect when applied as antimicrobial agents in lettuce^7^. Nevertheless, their commercial or industrial utilization has not been established yet, despite their reported antimicrobial activity and their low production cost^7^.

Although a significant amount of scientific work has been published so far, the industrial application of natural antimicrobials is one of the greatest challenges that food industry has to meet in the twenty-first century^33^. Seeking for new natural antimicrobials that could be used instead or in combination with low levels of essential oils to overcome the above limitations is of paramount importance towards this direction.

This study was motivated by the notion that the application of essential oil distillation by-products, apart from their ecological and eco-friendly character, can pose a significant antimicrobial activity without having the strong sensory effects of the essential oils. Nine laboratory rich-phenolic hydro-distilled aqueous extracts and one industrial hydrolate acquired as by-products of essential oil purification procedure were tested against three *S.* Typhimurium strains (4/74, FS8 and FS115). Τhe collected hydro-distilled extracts were odorless since the essential oil was removed and, thus, they can be used without compromising the sensory characteristics (odor and flavor) of the food products. Industrial hydrolate collected as by-product of steam distillation, the most commonly applied method of essential oil production^34^, can contain only a small amount of essential oils, that is usually discarded^35^. Although the odor of the hydrolate can vary, it is far from the strong scent of essential oils that can cause headache, eye and skin irritations, etc.^36^. According to the manufacturer, the industrial hydrolate used in the current study contained less than 0.5 ‰ of the essential oil compounds.

All of the extracts tested inhibited or reduced *Salmonella* strains when in vitro examined. However, the susceptibility of the strains was mainly affected by incubation temperature and plant extract. At 37 °C, incubation in the extracts caused either inactivation/reduction or growth inhibition of the strains. On the other hand, the combination of low temperature and antimicrobial compounds during incubation at 4 °C resulted in inactivation/reduction of all pathogens. A temperature-dependent effect regarding the antimicrobial activity of phenolic compounds has been previously reported^37^. In addition, a marked variability in the antimicrobial potential of the extracts was observed at both incubation temperatures. This may be attributed to variations of the phytocompounds present in the extracts^26,29,38^ as well as variations in their volatile nature (industrial hydrolate *vs* hydro-distilled extracts)^26,29^. For instance, a unique composition was observed for each one of the hydro-distilled extracts composed of phenolic compounds, even though rosmarinic acid was the most abundant component in half of the extracts tested. Nevertheless, these extracts had a milder in vitro antimicrobial activity compared to the industrial oregano hydrolate. The latter was composed of the volatile compounds thymol and carvacrol, the main components of oregano essential oil^39^. The superior antimicrobial effect of essential oils compared to their corresponding water extracts has also been reported elsewhere^40,41^. The absence of carvacrol and thymol from the hydro-distilled oregano and thyme extracts can be attributed to the volatilization of these compounds in the essential oil-fraction during the distillation process.

The antimicrobial effect of laurel extract at 37 °C on *Salmonella* was strain-dependent. Strain variations were also observed regarding the sensitivity of the strains inoculated in basil, calendula, c. oregano and corn silk at 4 °C. Miceli et al.^28^ also reported that the susceptibility of several strains of *Listeria monocytogenes*, *Salmonella enterica*, *Staphylococcus aureus* and *Enterobacter* spp. to two different plant water extracts was strain-specific. Food preservation methods or stresses widely occurring in the food chain can stimulate diverse strain-specific phenotypic responses. For instance, variations in the thermal^42^ or acid^42,43^ resistance has been reported among *Salmonella* strains. Gavriil et al.^44^ reported considerable variability regarding the innate resistance of six *Salmonella* strains to mayonnaise stored at 4 °C. According to Melo et al.^45^, three strains of *Listeria monocytogenes* adapted to a cheese stimulated medium used different proteomic repertoires in order to survive gastric stress, leading to a unique proteomic profile for each strain.

The in situ efficacy of both hydro-distilled oregano extract and industrial oregano hydrolate having the most prominent in vitro antimicrobial potency was further evaluated in pork meat under refrigeration (4 °C). Extract-based marination, edible coatings or their combinations supplemented without or with low concentrations of OEO were used as antimicrobial interventions for distributing the extracts onto the food surface. In accordance to the in vitro results, industrial oregano hydrolate was the most potent in reducing *Salmonella* populations on meat samples when applied in marinades or incorporated in edible films, though low levels of OEO were also required for the in situ antimicrobial activity to be manifested. Nevertheless, the type of the applied treatment (marination or edible coatings) affected the antimicrobial performance of the extract. Marination proved a rapid and effective intervention, even though no residual activity was observed during storage. Direct application of phytochemicals into the food system results in rapid diffusion of the antimicrobial agents into the mass of the food^23^. Application of edible coatings prepared with industrial oregano hydrolate + OEO, on the other hand, apart from a rapid reduction immediately after application, had also a gradual reduction during storage. Edible coatings are reported to allow a gradual and controlled diffusion of bioactive compounds into the food system^6,46^, maintaining, therefore, their concentrations on the surface of the foods at appropriate levels over time^46^. Application of a multi-hurdle approach combining both different antimicrobials and different interventions was required in the case of the hydro-distilled oregano extract that had showed lower in vitro antimicrobial activity to enhance its in situ antimicrobial performance. With regard to antagonistic effects that can take place when combining different chemical compounds^47^, this result demonstrate that rich phenolic by-products can effectively be combined with low levels of essential oils. The above in situ results are in line with other studies, demonstrating that the combination of natural antimicrobials or their use together with other preservation technologies may enhance their effectiveness^7,48,49^.

Nevertheless, the in situ activity of the extracts was lower compared to the in vitro assays. It is generally accepted that the antimicrobial effect of plant extracts may be markedly moderated in food substrates^27,28,49^. Food with a complex composition such as meat and poultry products can limit effective application of natural antimicrobials due to their inherent characteristics, such as their nonhomogeneous substrate, their neutral pH and their high concentrations of lipids and proteins^1^. Indeed, in complex matrices, lipids and proteins can strongly interact with bioactive phenolic compounds, lowering their antimicrobial efficacy^50^. Del Campo et al.^51^ demonstrated that the presence of serum albumin in TSB and dairy cream in TSB and zucchini broth reduced the inhibitory effect of a rosemary extract. Similarly, Bouarab-Chibane et al.^31^ reported that the antimicrobial activity of some phenolics against *Staphylococcus aureus* was reduced/abolished in the presence of bovine meat proteins, probably due to a decrease in the active free quantity of these antimicrobials. The level of the initial bacterial concentration has been also found to affect the antimicrobial profile of the extracts, with low levels of *Campylobacter jejuni* cell populations being more susceptible compared to high inocula levels^49^. Furthermore, the lower water content of food substrates compared to the laboratory media could also hinder the transfer of antimicrobial molecules into the active site within the microbial cell^52^. Regarding edible coatings, interactions of the forming biopolymer with the bioactive compounds may also affect the free residual concentration of the bioactive compounds. Gómez-Estaca et al.^53^ demonstrated reduced free phenol content in tuna-skin gelatin films as a result of polyphenol-protein interactions. Interactions of alginates with polyphenols has also been reported^54^. The effectiveness of the aqueous extracts other than essential oils in reducing pathogen load in foods has been previously reported, with regard to factors such as food matrix, storage temperature and type and concentration of plant extract^7,12,37,55–57^.

Despite the limited (on average), yet significant in situ reductions caused by the antimicrobial interventions studied, they are considered of great importance in the field of food safety, especially when it comes to substances with a milder antimicrobial nature compared to essential oils. The results of the current study highlight the potential of these extracts as antimicrobial food agents and leave room for further studying new ways that could maximize their antimicrobial performance. The concentrations of essential oils in food products required for sufficient antimicrobial activity usually exceeds their organoleptically acceptable levels^58^. Therefore, their combination with plant extracts could minimize the required dose^59^, leading to acceptable sensory properties of treated products.

In conclusion, the aqueous by-products of essential oil production effectively controlled *S.* Typhimurium in a temperature-, plant- and strain-dependent manner when in vitro examined. Oregano essential oil by-products exhibited prominent bactericidal properties against FS8 strain inoculated on pork meat at 4 °C. Despite their mild antimicrobial activity when applied in situ, the utilization of these extracts in combination with low levels of essential oils can adequately control the safety of pork meat when applied in the concept of a multi-hurdle approach, in an eco-friendly and cost-effective way, without compromising the sensory properties (odor and flavor) of the food products. Future work could focus on finding new ways for maximizing their antimicrobial performance, e.g. combining hydro-distilled extracts with different chemical composition in a variety of food products. Furthermore, future studies might also unravel the mechanisms behind the strain-dependent effects that were observed in this study by exploring the proteomic profiles of the strains, during exposure to the class of antimicrobials tested.

## Methods

### Bacterial strains and inoculum preparation

Ten plant aqueous extracts were screened for their in vitro antimicrobial profile against three different strains of *Salmonella enterica* subspecies *enterica* serovar Typhimurium (strains 4/74, FS8 and FS115). Strain 4/74 isolated from calf bowlel was kindly provided by the Laboratory of Food Microbiology and Biotechnology, Agricultural University of Athens, Greece whereas strains FS8 and FS115 were isolated from meat samples (pork minced meat and chicken meat, respectively) collected from a food industry. Stock cultures were maintained in Tryptone Soy Broth (TSB, Lab M Limited, Lancashire, UK) at − 20 °C supplemented with 20% glycerol and were monthly subcultured to Tryptic Soy Agar (TSA, Lab M Limited, Lancashire, UK) stored at 0–4 °C. Before every experiment, one colony from each strain was transferred into 10 ml TSB (37 °C, 24 h), followed by a second transfer of 100 μl from 24-h cultures to 10 ml of fresh TSB (37 °C, 18 h). Before their use, activated cells were centrifuged (2709×*g*, 10 min, 4 °C), washed twice with ¼ Ringer solution (Lab M Limited, Lancashire, UK) and resuspended in 10 ml of the appropriate medium (aqueous extracts, de-ionized sterile water, TSB, TSB adjusted to pH 5.5 or 3.5), as described below. Antimicrobial activity of hydro-distilled oregano extract and industrial oregano hydrolate in pork meat was evaluated using only FS8 strain. The strain was prepared as described above and resuspended in 10 ml of ¼ Ringer solution.

### Extraction of aqueous plant extracts

Basil, calendula, corn silk, laurel, oregano, rosemary, spearmint and thyme were purchased in a dried form from a local supermarket. Each plant was mixed with de-ionized water at a 1:10 ratio and hydro-distilled in a Clevenger apparatus for 3 h, according to Gardeli et al.^60^. After the end of hydro-distillation, both essential oil and hydrolate were discarded, whereas the remaining liquid waste where the plant was boiled was collected by removing the solid retentate. Centrifuged oregano extract was collected by centrifuging the hydro-distilled oregano extract (2709×*g*, 5 min, 4 °C) and using the supernatant as a new antimicrobial aqueous phase. All the aqueous fractions were covered with silver foil and stored at 4 °C for 48 h before further use.

An industrially acquired oregano hydrolate collected as by-product of oregano essential oil steam distillation was also used throughout this study. The aqueous extract was purchased by an industry in Northern Greece and was transferred to the laboratory. Steam distillation was performed for at least 3 h. According to the manufacturer, the hydrolate contained less than 0.5 ‰ of the essential oil compounds. The extract was covered in silver foil and stored at 4 °C until use.

### In vitro antimicrobial activity of plant extracts

The activated cultures of *Salmonella* strains were serially diluted and inoculated in the appropriate medium (aqueous extracts, de-ionized sterile water, TSB, TSB adjusted to pH 5.5 or 3.5 with HCl 6 N; Merck, Darmstadt, Germany) at a final concentration of approximately 10^6^ CFU/ml. De-ionized water and TSB were used as controls. Inoculation in TSB adjusted to pH 5.5 or 3.5 (HCl 6 N) was performed in order to simulate the lower pH values of hydro-distilled extracts (pH 5.2–5.6) and industrial oregano hydrolate (pH 3.5), respectively. The inoculated solutions were incubated at either 37 or 4 °C; sampling was performed at 0, 3, 6, 9 and 24 h or at regular time intervals for up to 44 days (depending on the plant extract), respectively. Enumeration was carried out by plating 50 μl of the appropriate dilution on TSA supplemented with 0.1% sodium pyruvate (AppliChem, Lot 600104 67) (TSA/SP). The detection limit was set at 1.3 log CFU/ml. Petri dishes were incubated at 37 °C for 48 h.

All experiments were conducted in triplicate in two independent replicates.

### Preparation of antimicrobial edible coatings

Edible coatings made of sodium alginate 1.5% w/v were prepared using water, hydro-distilled oregano extract and industrial oregano hydrolate. For the preparation of the coatings, appropriate quantity of the sodium alginate base was added to the extracts, previously heated at 50–60 °C, and stirred until its total dissolution. Coatings prepared of hydro-distilled oregano extract did not form the film matrix when immersed to CaCl_2_. Therefore, preparation of these coatings was performed either by mixing the extract with de-ionized sterile water in a ratio 1:1 (50% oregano), by using centrifuged oregano extract as the film forming material or by using their combination (i.e. centrifuged oregano extract diluted 1:1 in de-ionized sterile water; 50% centrifuged oregano). The ratio 1:1 was selected after several trials as the only proportion of sterile water and hydro-distilled oregano extract that enabled the formation of the coatings without minimizing the quantity of the oregano extract. Industrial oregano hydrolate was also diluted 1:1 in de-ionized water (50% industrial) in order to further compare their activity. Coatings prepared of de-ionized water were used as control. All coatings were supplemented with or without 0.2 or 0.5% v/v oregano essential oil (OEO). Incorporation of OEO into the extract alginate solutions was performed using an Ultra Turrax (5 min, 26,000 rpm) under aseptic conditions. In summary, the coatings used were the followings: de-ionized sterile water, de-ionized sterile water + OEO, 50% v/v oregano, 50% v/v oregano + OEO, centrifuged oregano, centrifuged oregano + OEO, 50% v/v centrifuged oregano, 50% v/v centrifuged + OEO, industrial oregano, industrial oregano + OEO, 50% v/v industrial oregano, 50% v/v industrial oregano + OEO.

### Meat preparation and inoculation

Pork meat thigh was purchased from a local supermarket and transferred to the laboratory within 20 min. Pieces of 10 ± 0.2 g were aseptically cut and stored at 4 °C until their use. For their inoculation, 100 μl of the culture were gently spread on the entire upper surface of the sample and allowed to adhere for 15 min at 4 °C. The same process was carried out for the bottom surface of the samples, followed again by a fifteen-minute adhesion of the inoculum. The final cell concentration of *Salmonella* on the meat was approximately 10^6^ CFU/g.

### Εvaluation of oregano aqueous extracts antimicrobial activity against *S.* Typhimurium FS8 on pork meat

After their inoculation with *S.* Typhimurium FS8, pork meat samples were subjected to three types of antimicrobial treatments, all based on hydro-distilled oregano extract and industrial oregano hydrolate: (a) marination, (b) edible coatings, (c) combined marination and edible coatings.

#### Evaluation of marination antimicrobial activity against *S.* Typhimurium FS8 on pork meat

Hydro-distilled oregano extract, industrial oregano hydrolate and de-ionized sterile water (control) supplemented with or without 0.2% oregano essential oil were used as marinating solutions and tested for their antimicrobial activity against FS8 strain. Inoculated pork samples were immerged in 300 ml of each solution and marinated for 3 h at 25 °C (room temperature). Samples were, then, placed in capped petri dishes and stored at 4 °C for 4 days. Sampling was performed immediately after treatment and after 4 days of storage.

#### Evaluation of edible coatings antimicrobial activity against *S.* Typhimurium FS8 on pork meat

Sodium alginate (1.5% w/v) edible coatings of de-ionized sterile water, de-ionized sterile water + 0.5% OEO, 50% oregano, 50% oregano + 0.5% OEO, centrifuged oregano, centrifuged oregano + 0.5% OEO, 50% centrifuged oregano, 50% centrifuged + 0.5% OEO, industrial oregano, industrial oregano + 0.5% OEO, 50% industrial oregano, 50% industrial oregano + 0.5% OEO were prepared as described above. Inoculated pork samples were first immersed in the coating solution for 2 min, allowed to drain for 2–3 s and then dipped into calcium chloride (CaCl_2_) solution (2% w/v) for 3 min. Finally, they were placed in capped petri dishes and stored at 4 °C for 5 days. Sampling was performed immediately after coating and once per day for up to 5 days of storage.

#### Evaluation of combined marination and edible coatings antimicrobial activity against *S.* Typhimurium FS8 on pork meat

Inoculated pork meat samples were marinated with hydro-distilled oregano extract, industrial oregano hydrolate or de-ionized sterile water supplemented with or without 0.2% OEO as described above and then covered with sodium alginate (1.5% w/v) edible coatings made of de-ionized sterile water, de-ionized sterile water + 0.2% OEO, 50% oregano, 50% oregano + 0.2% OEO, industrial oregano, industrial oregano + 0.2% OEO, as described above. Samples were finally placed in capped petri dishes and stored at 4 °C for 4 days. Sampling was performed immediately after marination and on the 2nd and 4th day of storage.

In each of the above experiments, uninoculated untreated samples and inoculated untreated samples were used as controls. Experiments were performed in duplicate in three independent replicates.

### Microbiological analysis

Microbiological analysis of pork samples was performed by homogenizing each sample (10 ± 0.2 g) with 90 ml ¼ Ringer solution in a stomacher apparatus (Seward, London, UK) for 60 s. Enumeration of surviving *Salmonella* populations was performed by plating 100 μl of the appropriate dilution in Xylose Lysine Decarboxylase (XLD; Lab M Limited, Lancashire, UK). Plates were incubated for 24 h at 37 °C.

### pH measurement

Changes in the pH of pork samples were determined at the end of microbiological analysis by immersing the electrode of a digital pHmeter (pH 526, Metrohm Ltd, Switzerland) in the homogenized pork samples.

### Composition of the aqueous extracts

The chemical composition of hydro-distilled aqueous extracts and industrial oregano hydrolate was determined by Ultra High Performance Liquid Chromatography Heated Electrospray Ionization—Tandem Mass Spectrometry (UPLC-HESI-MS/MS) and High Performance Liquid Chromatography coupled to Diode Array detector (HPLC–DAD), respectively, as described in details in supplementary data (see Supplementary Methods online).

### Determination of inactivation parameters

Determination of the time needed for a 4-log reduction (t_4D_) was determined for the in vitro data at 4 °C by fitting the log transformed populations to Weibull model according to the equation log*N/No* = − (t/*δ*)^p^^61^, where N_o_ the populations at time t_o_, N the population at time t, N_res_ the residual bacterial concentration log CFU/ml at the end of microbial inactivation, *δ* the time needed for the first decimal reduction and *p* a shape parameter corresponding to different concavities (downward concave survival for curves *p* > 1, upward concave survival curves for *p* < 1 or linear curves for *p* = 1). GinaFit, a freeware Add-ins for Microsoft^®^Excel^62^ was used for data fitting. In total, six curves per experimental case were fitted.

### Statistical analysis

Analysis of Variance (SPSS 22.0 for Mac) was used for data analysis of the log transformed cell populations collected from the in vitro screening at 37 °C and the in situ treatments and for the t_4D_ estimates of the in vitro screening at 4 °C. Means were compared using Tukey’s Honestly Significant Test (HSD). For pairwise comparisons, *t-test* (Microsoft Excel 16 for Mac) was used. Differences were considered significant at 95% level.

## Supplementary Information

Supplementary Information.

## Data Availability

All data generated or analyzed during this study are included in this published article (and its Supplementary Information files).
