# Supplementary material for: Evaluation of antimicrobial activities of plant aqueous extracts against Salmonella Typhimurium and their application to improve safety of pork meat
Source: Sci Rep. 2021 Nov 9;11:21971. doi: 10.1038/s41598-021-01251-0 (PMC8578650; doi:10.1038/s41598-021-01251-0)
Supplement: Supplementary file 1 — Supplementary Information. [file 41598_2021_1251_MOESM1_ESM.pdf]

Evaluation of antimicrobial activities of plant aqueous extracts against *Salmonella* Typhimurium and their application to improve safety of pork meat

Supplementary Methods

Composition of the aqueous extracts

- Chemical standards and solvents

Chemical standards were purchased from Extrasynthese (Genay Cedec, France), Sigma-Aldrich (St. Louis, MO, USA), Alfa Aesar (Haverhill, MA, USA) and Fluka Chemie (Buchs, Switzerland) with purity above 90%. LC-MS grade solvents and analytical grade ethanol were obtained from Merck (Darmstadt, Germany). For the chromatographic analysis HPLC-grade water was prepared using a Milli-Q system (Merck Millipore, Burlington, MA, USA). Prior to use, all solvents (except acetonitrile) were filtered through cellulose acetate membranes of 0.45 μm pore size.

- Stock Solutions

The stock solutions of samples and analytical standards were prepared by dissolution in methanol at concentration of 1000 μg/ml (in limited cases the solubility was enhanced with the addition of low amount of dimethylsulfoxide, DMSO) and maintained at -18 °C in the absence of light. These solutions were diluted and utilized for the construction of calibration curves immediately prior to the performance of the analysis. For each sample, duplicate analyses were carried out by direct injection into the analytical instruments.

- Preparation of hydro-distilled aqueous extracts

The hydro-distilled aqueous extracts, obtained as described the main text (see Materials and Methods), were freeze-dried (Scientz-18N, Freeze dryer, Ningbo Scientz Biotechnology Co., Ltd., China) to provide the respective samples as the dry powders that were subsequently subjected to chemical analysis.

- Preparation of steam-distilled aqueous extract (industrial oregano hydrolate)

The sample of steam-distilled industrial oregano hydrolate, was prepared by mixing 35 ml of the extract with an equal quantity of pentane (Carlo Ebra Reagents, Val de Reuil, France) and agitated until all gases were removed. This procedure was repeated twice and the combined organic phases were removed using a rotary evaporator (Rotavapor R-210, Büchi, Flawil, Switzerland).

- Analytical Method Validation

The validation of the analytical method developed was performed through the construction of calibration curves utilizing at least 6 points (with 3 replicates per level) for every analyte. The linear calibration curves in the studied concentration range of 10 to 2000 ng/ml, demonstrated acceptable correlation coefficient values ($r^2 \geq 0.99$) for every analyte. Recovery of the investigated compounds (as a criterion of the trueness of the method) was evaluated at two concentration levels (40 and 400 ppb) by the addition of mixed solutions of the standards into the respective extract and were within the acceptable range of 80%−120%. Considering that the sampled plant material (oregano, thyme, rosemary etc.) contains numerous of the studied analytes, the method validation was verified by utilizing the standard addition procedure. Precision values were always acceptable with percent Relative Standard Deviation (RSD%) < 14%.

- Determination of bioactive phytochemicals content of hydro-distilled aqueous extracts using UPLC-HESI-MS/MS

The Ultra High Performance Liquid Chromatography Heated Electrospray Ionization - Tandem Mass Spectrometry (UPLC-HESI-MS/MS) analysis were carried out on an Accela Ultra High-Performance Liquid Chromatography system (Thermo Fisher Scientific, Waltham, MA, USA), coupled with a TSQ Quantum Access triple quadrupole mass spectrometer operated in multiple reaction monitoring (MRM) mode and equipped with an autosampler (Thermo Fischer Scientific, San Jose, CA, USA). Mass spectrometric analysis was conducted using heated electrospray ionization (HESI), operated in two complementary modes (positive and negative). Additionally, the Selected Ion Monitoring (SIM) mode was adequate and used to confirm the presence of several analytes. Ion source and vacuum parameters of the mass spectrometer, precursor and product ions and the collision activated ionization for the target analytes, were obtained-optimized by the direct infusion in full scan mode of their standard solutions. The spray voltage was set at 2700 V, sheath gas (nitrogen) and auxiliary gas (argon) pressures were set at 25 and 10 arbitrary units, respectively. Capillary temperature was set at 320 °C and collision pressure at 1.5 mTorr. The monitoring ion transitions are presented in the Table below. Phytochemicals were separated on a Hypersil Gold 3 μm, 100×2.1mm i.d. chromatographic column (Thermo Fischer Scientific, San Jos, CA), using a a flow rate of 300μl/min, and a mobile phase consisting of: water (A) and acetonitrile (B), both containing formic acid (0.1%). The gradient program was: 0.0–1.0 min: 10% B, 1.0–12.0 min from 10% B to 100%, 12.0-12.1 min 10%B, and 12.1–14.0 min 10% B. The injection volume was 10 μL, maintaining a column temperature at 35 °C.

Table: MRM transitions and the utilized respective parameters

Compound	Precursor ion (m/z)	Quantitation ion, m/z (collision energy, eV)	Confirmation ion(s), m/z (collision energy, eV)	Ionization mode
Rutin	609	301 (15)	271 (21)	(-)
Quercetin	300,9	179 (20)	151 (20)	(-)
Chlorogenic acid	353.1	191 (15)		(-)
Syringic acid	197.1	179 (18)	135 (15)	(-)
Naringenin	271	151 (21)	253 (20)	(-)
Ellagic acid	300.8	283.8 (30)	228.9 (28)	(-)
Caffeic acid	181	178 (12)	134 (14)	(+)
Rosmarinic acid	359	161 (18)	197 (15)	(-)
Luteolin	285	151 (24)	199 (20), 132,8 (27)	(-)
Apigenin	269	117 (30)	151 (30)	(-)
Hesperidin	610.9	303.1 (21)	465.1 (17)	(+)
Kaempferol	285	255 (28)	117 (28)	(-)
Gallic acid	168.9	125.2 (12)	79.2 (17)	(-)
Pinocembrin	256.1	152.9 (21)		(+)
p-Coumaric acid	163	119 (20)	93 (22)	(-)
Myricetin	317	179.2 (23)	151.3 (24)	(-)
Orientin	447.2	327 (19)		(-)
Catechin	288.3	109 (25)		(-)
Vitexin	431	311 (18)		(-)
Hyperoside	463.1	300.1 (16)		(-)
Diosmin	609	463 (21)		(+)
Phloridzin	435	167 (28)		(-)
Adipic acid	145.1	101.1 (20)		(-)

- HPLC-DAD method for the determination of steam-distilled industrial hydrolate

The carvacrol and thymol content of industrial oregano hydrolate was determined by High Performance Liquid Chromatography coupled to Diode Array detector (HPLC-DAD). The analysis was performed on an Agilent 1100 instrument (Agilent Technologies, Santa Clara, CA, USA) equipped with a diode-array detector (measuring absorbance over the full wavelength range during the entire run). The column used was a Kromasil C18 column (250mm x 4.6mm, particle size 5 μm), thermostated at 30 °C, and connected with a guard column of the same material (8mm x 4 mm). Injection was performed through a Rheodyne injection valve (model 7725I) with a 20 μl fixed loop. For the chromatographic analysis HPLC-

grade water was prepared using a Milli-Q system (Merck Millipore, Burlington, MA, USA), whereas all HPLC grade solvents (except acetonitrile) were filtered prior to use through cellulose acetate membranes of 0.45 μm pore size. The mobile phase consisted of solvent A (obtained by the addition of 3% acetic acid in 2 mM sodium acetate aqueous solution) and solvent B (acetonitrile). Run time was set at 70 min with a constant flow rate at 1.0 ml/min in accordance with the following gradient: 0 min, 95% A and 5% B; after 45 min, the pumps were adjusted to 85% A and 15% B; at 60 min, 65% A and 35% B; at 65 min, 50% A and 50% B; and finally, at 70 min, 100% B. This routine was followed by a 30 min equilibration period. Peaks were identified by comparing their retention times and UV–vis spectra with the reference compounds, and data were quantitated using the calibration curves obtained for the reference analytes. HP ChemStation 5.01 software package system (Hewlett Packard, Palo Alto, CA, USA) was used for data acquisition and analysis.

Supplementary Figures

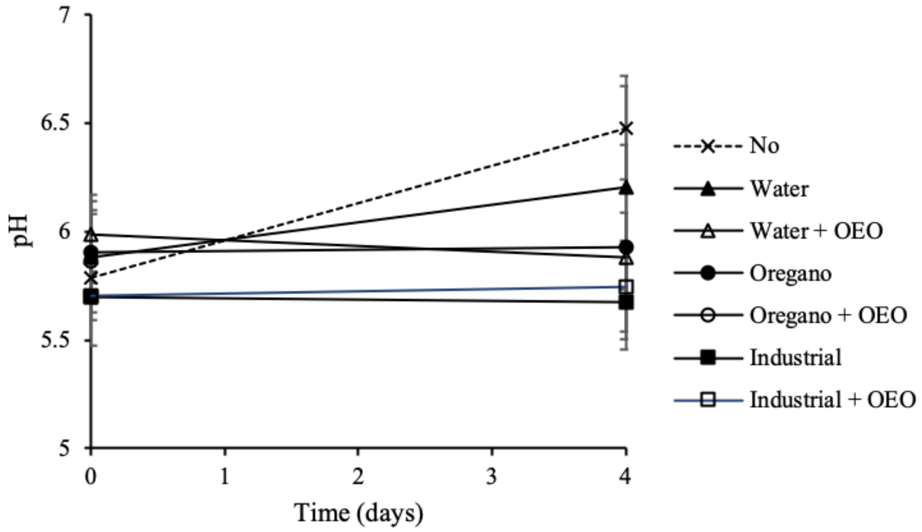

Supplementary Fig. S1: pH changes of inoculated pork meat samples during storage at 4ºC following 3-h marination to water, hydrodistilled oregano extract or industrial oregano hydrolate supplemented with or without 0.2% OEO. Each data point is an average (± standard deviation) of six replicates.

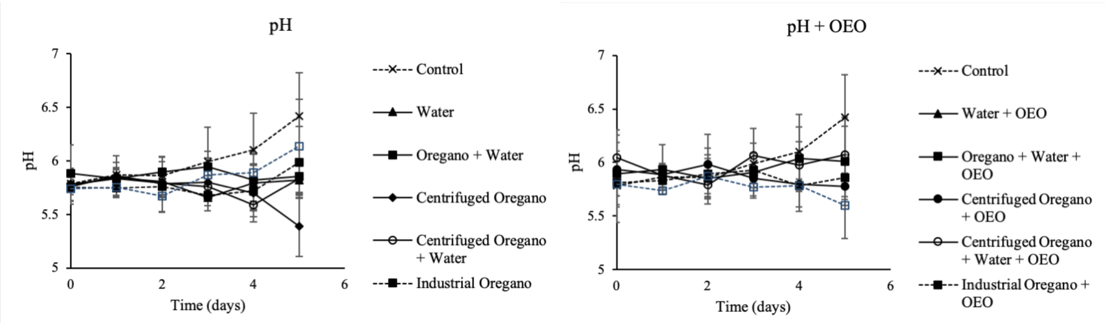

Supplementary Fig. S2: pH changes of inoculated pork meat samples coated with water, hydro-distilled oregano extract or industrial oregano hydrolate supplemented with or without 0.5% OEO during storage at 4ºC. Each data point is an average (± standard deviation) of six replicates.

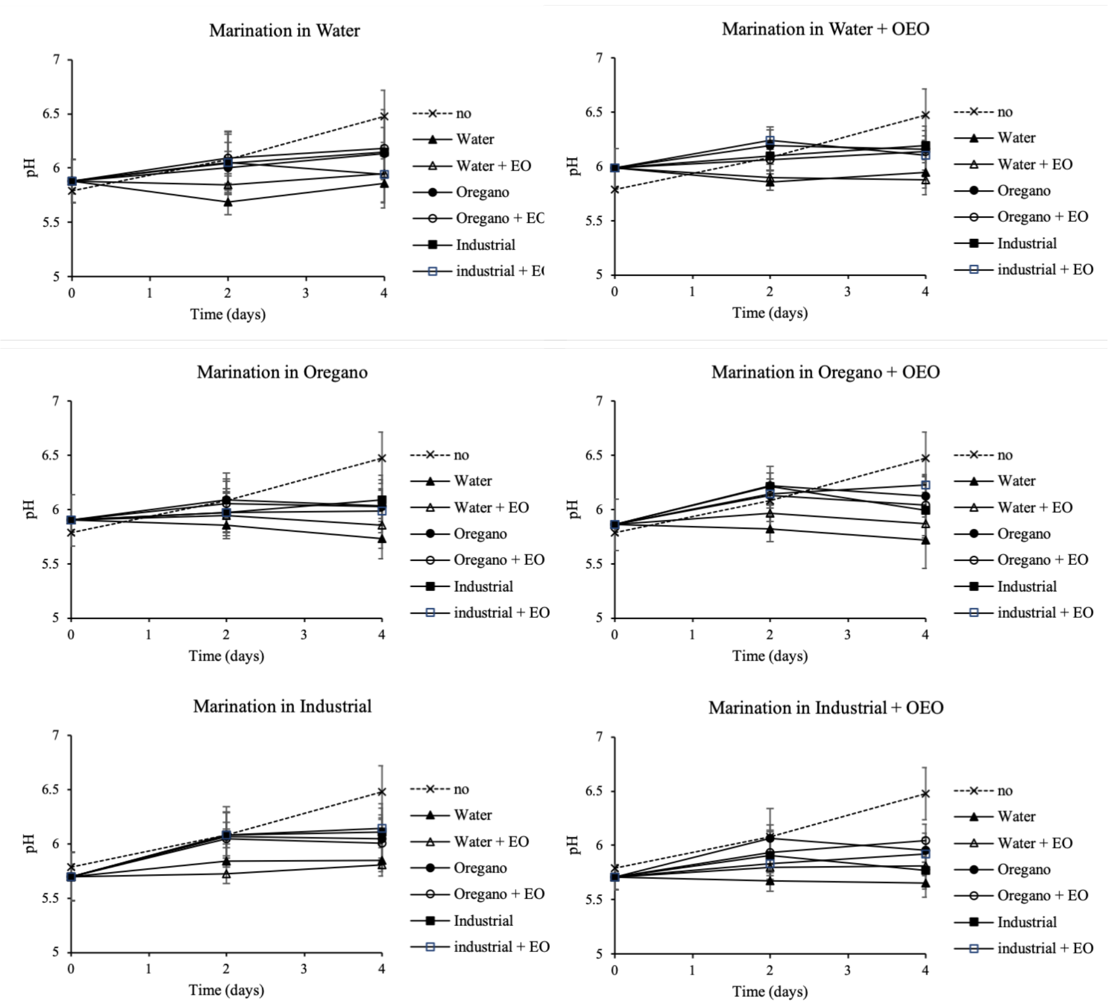

Supplementary Fig. S3: pH changes of inoculated pork meat samples marinated in water, hydro-distilled oregano extract or industrial oregano hydrolate supplemented with or without 0.2% OEO and coated with water, hydro-distilled oregano extract or industrial oregano hydrolate supplemented with or without 0.2% OEO at 4ºC. Each diagram represents samples marinated in the same marinating solution, whereas lines within each diagram samples coated with a different forming material. Each data point is a mean of 6 replicates (± standard deviation).

Supplementary Tables

Supplementary Table S1: *In vitro* antimicrobial effect of TSB adjusted to pH 3.5 on *S.* Typhimurium strains at 4 ºC and 37ºC

Temperature	Time (h)	4/74	FS8	FS115
4ºC	0	5.7 ± 0.1	5.8 ± 0.1	5.8 ± 0.1
	3	5.7 ± 0.1	5.7 ± 0.1	5.7 ± 0.1
	6	5.5 ± 0.2	5.5 ± 0.1	5.4 ± 0.2
	9	5.3 ± 0.1	5.4 ± 0.1	5.3 ± 0.1
	24	4.7 ± 0.1	4.9 ± 0.1	4.7 ± 0.1
37ºC	0	5.7 ± 0.1	5.7 ± 0.1	5.8 ± 0.1
	1.5	5.4 ± 0.1	5.4 ± 0.1	5.4 ± 0.1
	3	4.8 ± 0.1	4.8 ± 0.1	4.8 ± 0.1
	6	3.5 ± 0.1	3.9 ± 0.2	3.5 ± 0.1
	9	2.3 ± 0.5	3.0 ± 0.1	2.8 ± 0.1

Supplementary Table S2: *In vitro* antimicrobial effect of hydro-distilled aqueous plant extracts on *S.* Typhimurium strains at 37°C

	4/74					FS8					FS115				
	**0 h**	**3 h**	**6 h**	**9 h**	**24 h**	**0 h**	**3 h**	**6 h**	**9 h**	**24 h**	**0 h**	**3 h**	**6 h**	**9 h**	**24 h**
Basil	5.5 ± 0.3 **(A, cd)**	5.5 ± 0.3 **(A, de)**	5.4 ± 0.2 **(A, de)**	5.2 ± 0.3 **(B, f)**	1.8 ± 1.2 **(A, f)**	5.3 ± 0.3 **(A, cd)**	5.3 ± 0.2 **(A, ef)**	5.3 ± 0.2 **(A, de)**	5.4 ± 0.2 **(A, d)**	2.1 ± 1.4 **(A, f)**	5.5 ± 0.3 **(A, cd)**	5.5 ± 0.3 **(A, de)**	5.5 ± 0.2 **(A, e)**	5.5 ± 0.1 **(A, de)**	2.1 ± 1.4 **(A, f)**
Calendula	5.8 ± 0.2 **(A, ab)**	5.3 ± 0.2 **(A, e)**	3.7 ± 0.1 **(B, h)**	3.3 ± 0.1 **(B, h)**	1.0 ± 0.0 **(A, g)**	5.8 ± 0.1 **(A, a)**	5.4 ± 0.1 **(A, de)**	3.9 ± 0.1 **(A, g)**	3.6 ± 0.1 **(A, f)**	1.0 ± 0.0 **(A, g)**	5.9 ± 0.1 **(A, a)**	5.4 ± 0.1 **(A, e)**	4.0 ± 0.2 **(A, h)**	3.3 ± 0.1 **(B, g)**	1.1 ± 0.1 **(A, g)**
C. oregano	5.7 ± 0.3 **(A, abc)**	5.4 ± 0.3 **(A, de)**	4.7 ± 0.3 **(A, fg)**	3.9 ± 0.9 **(A, g)**	1.0 ± 0.1 **(B, g)**	5.7 ± 0.3 **(A, ab)**	5.5 ± 0.4 **(A, de)**	4.7 ± 0.4 **(A, f)**	4.3 ± 0.9 **(A, e)**	1.4 ± 0.4 **(A, fg)**	5.7 ± 0.2 **(A, ab)**	5.5 ± 0.3 **(A, de)**	5.0 ± 0.3 **(A, fg)**	4.2 ± 0.9 **(A, f)**	1.0 ± 0.1 **(B, g)**
Corn silk	5.8 ± 0.1 **(A, ab)**	5.7 ± 0.1 **(A, cde)**	6.4 ± 0.1 **(C, c)**	7.5 ± 0.2 **(AB, c)**	7.6 ± 0.2 **(A, b)**	5.7 ± 0.1 **(AB, ab)**	5.7 ± 0.2 **(A, cd)**	6.6 ± 0.2 **(B, c)**	7.4 ± 0.1 **(B, c)**	7.7 ± 0.1 **(A, b)**	5.7 ± 0.1 **(B,ab)**	5.7 ± 0.1 **(A, cd)**	7.0 ± 0.1 **(A, c)**	7.5 ± 0.1 **(A, c)**	7.5 ± 0.1 **(A, b)**
Laurel	5.6 ± 0.2 **(A, bc)**	5.6 ± 0.2 **(A, cde)**	5.5 ± 0.1 **(A, de)**	5.4 ± 0.1 **(B, f)**	3.3 ± 0.5 **(C, e)**	5.6 ± 0.2 **(A, bc)**	5.6 ± 0.1 **(A, cd)**	5.5 ± 0.2 **(A, de)**	5.6 ± 0.1 **(A, d)**	6.8 ± 0.3 **(A, d)**	5.6 ± 0.2 **(A, bc)**	5.6 ± 0.2 **(A, cde)**	5.5 ± 0.2 **(A, de)**	5.5 ± 0.2 **(AB, de)**	4.1 ± 1.0 **(B, e)**
Oregano	5.9 ± 0.2 **(A, a)**	4.5 ± 0.6 **(A, f)**	1.3 ± 0.5 **(A, i)**	1.0 ± 0.2 **(AB, i)**	1.0 ± 0.0 **(g)**	5.9 ± 0.2 **(A, a)**	4.7 ± 0.4 **(A, g)**	1.8 ± 0.6 **(A, h)**	1.0 ± 0.0 **(B, g)**	1.0 ± 0.0 **(g)**	5.9 ± 0.2 **(A, a)**	4.9 ± 0.5 **(A, f)**	1.8 ± 0.9 **(A, i)**	1.3 ± 0.7 **(A, h)**	1.0 ± 0.0 **(g)**
Rosemary	5.3 ± 0.1 **(A, d)**	5.3 ± 0.2 **(A, e)**	5.2 ± 0.1 **(AB, ef)**	5.3 ± 0.2 **(A, f)**	6.9 ± 0.1 **(A, c)**	5.2 ± 0.1 **(B, d)**	5.1 ± 0.1 **(B, f)**	5.1 ± 0.1 **(B, ef)**	5.4 ± 0.2 **(A, d)**	7.0 ± 0.1 **(A, cd)**	5.3 ± 0.1 **(AB, d)**	5.4 ± 0.1 **(A, e)**	5.3 ± 0.1 **(A, ef)**	5.2 ± 0.1 **(A, e)**	6.5 ± 0.2 **(B, c)**
Spearmint	5.6 ± 0.2 **(A, bc)**	5.5 ± 0.1 **(A, cde)**	5.7 ± 0.1 **(B, d)**	6.8 ± 0.1 **(C, d)**	7.3 ± 0.2 **(AB, bc)**	5.7 ± 0.1 **(A, ab)**	5.5 ± 0.2 **(A, cde)**	6.5 ± 0.4 **(A, c)**	7.4 ± 0.2 **(A, c)**	7.4 ± 0.1 **(A, bc)**	5.7 ± 0.2 **(A, ab)**	5.6 ± 0.1 **(A, de)**	5.9 ± 0.2 **(B, d)**	7.1 ± 0.2 **(B, c)**	7.1 ± 0.2 **(B, bc)**
Thyme	5.8 ± 0.1 **(A, ab)**	5.8 ± 0.3 **(A, cd)**	4.4 ± 0.9 **(A, g)**	4.0 ± 0.6 **(A, g)**	1.0 ± 0.1 **(A, g)**	5.7 ± 0.2 **(B, ab)**	5.6 ± 0.2 **(A, cde)**	4.7 ± 0.3 **(A, f)**	4.5 ± 0.4 **(A, e)**	1.0 ± 0.1 **(A, g)**	5.8 ± 0.1 **(AB, ab)**	5.6 ± 0.2 **(A, de)**	4.8 ± 0.2 **(A, g)**	4.5 ± 0.3 **(A, f)**	1.0 ± 0.0 **(A, g)**

TSB	5.7 ± 0.2 **(A, abc)**	7. 8± 0.2 **(A, a)**	9.2 ± 0.4 **(A, a)**	9.5 ± 0.2 **(A, a)**	9.5 ± 0.3 **(A, a)**	5.7 ± 0.3 **(A, ab)**	7.9 ± 0.2 **(A, a)**	9.0 ± 0.5 **(A, a)**	9.3 ± 0.4 **(B, a)**	9.5 ± 0.3 **(A, a)**	5.7 ± 0.2 **(A, ab)**	7.8 ± 0.2 **(A, a)**	9.1 ± 0.4 **(A, a)**	9.5 ± 0.2 **(A, a)**	9.5 ± 0.3 **(A, a)**
TSB pH 5.5	5.8 ± 0.1 **(A, ab)**	6.8 ± 0.1 **(A, b)**	8.2 ± 0.2 **(B, b)**	8.7 ± 0.2 **(A, b)**	9.2 ± 0.2 **(A, a)**	5.8 ± 0.1 **(A, ab)**	6.9 ± 0.1 **(A, b)**	8.2 ± 0.3 **(B, b)**	8.7 ± 0.1 **(A, b)**	9.2 ± 0.0 **(A, a)**	5.8 ± 0.1 **(A, ab)**	6.9 ± 0.2 **(A, b)**	8.5 ± 0.1 **(A, b)**	8.7 ± 0.1 **(A, b)**	9.2 ± 0.1 **(A, a)**
Water	5.7 ± 0.2 **(A, abc)**	5.9 ± 0.1 **(A, c)**	5.6 ± 0.2 **(A, d)**	5.9 ± 0.1 **(A, e)**	5.7 ± 0.2 **(A, d)**	5.7 ± 0.2 **(A, ab)**	5.8 ± 0.1 **(A, c)**	5.6 ± 0.2 **(A, d)**	5.6 ± 0.3 **(B, d)**	5.6 ± 0.3 **(A, e)**	5.7 ± 0.2 **(A, abc)**	5.9 ± 0.1 **(A, c)**	5.7 ± 0.2 **(A, de)**	5.8 ± 0.1 **(AB, d)**	5.6 ± 0.3 **(A, d)**

Different capital letters within the same row indicate statistical differences ($P < 0.05$) among the three strains inoculated to the same plant extract and for the same time interval according to Tukey's HSD

Different lowercase letters within the same column indicate statistical differences ($P < 0.05$) of a single strain inoculated to the different plant extracts according to Tukey's HSD

Supplementary Table S3: Order of antimicrobial potential of the hydro-distilled aqueous extracts at 4°C based on their t_{4D} estimates

Salmonella Typhimurium strains	Order of antimicrobial potential
S. Typhimurium 4/74	Oregano > Thyme > Calendula > C. oregano > Spearmint = Rosemary > Basil > Laurel > Corn silk
S. Typhimurium FS8	Oregano > Thyme > Calendula > C. oregano > Spearmint ≥ Rosemary = Basil > Laurel > Corn silk
S. Typhimurium FS115	Oregano > Thyme = Calendula > C. oregano ≥ Spearmint = Rosemary > Basil >Laurel > Corn silk

Supplementary Table S4: Effect of 3-hour marination treatment in the inactivation of *S.* Typhimurium FS8 inoculated in pork meat and stored at 4ºC. Untreated pork meat samples inoculated with the pathogen were used as controls

Time (days)	Extracts						
	Control	Water	Water + OEO	Oregano	Oregano + OEO	Industrial	Industrial + OEO
0	6.5 ± 0.2 **A, a**	5.3 ± 0.7 **BCD, a**	4.9 ± 0.5 **CD, a**	5.9 ± 0.5 **B, a**	5.4 ± 0.3 **BC, a**	4.7 ± 0.4 **DE, a**	4.1 ± 0.6 **E, a**
4	6.3 ± 0.2 **A, a**	6.0 ± 0.4 **A, b**	4.3 ± 0 1.1 **BC, a**	6.1 ± 0.2 **A, a**	4..9 ± 0.7 **B, a**	4.4 ± 0.4 **BC, a**	3.8 ± 1.0 **C, a**

Different capital letters within the same row indicate statistical differences ($P < 0.05$) according to Tukey's HSD

Different lowercase letters within the column indicate statistical differences ($P < 0.05$) according to *t-test*

Supplementary Table S5: Effect of edible coatings prepared of water, hydro-distilled oregano extract and industrial oregano hydrolate on the survival of *S.* Typhimurium FS8 inoculated in pork meat at 4ºC. Inoculated uncoated pork meat samples were used as controls. Sampling was performed immediately after treatment and up to 5 days of storage. Each value represents an average (± standard deviation) of six replicates

				**Extracts**			
**Time (days)**	**Control**	**Water**	**50% Oregano**	**Centrifuged oregano**	**50% Centrifuged oregano**	**Industrial oregano**	**50% Industrial oregano**
0	6.5 ± 0.2 **A, a**	6.1 ± 0.2 **B, a**	6.2 ± 0.1 **AB, a**	6.2 ± 0.2 **AB, a**	6.3 ± 0.2 **AB, a**	6.1 ± 0.3 **B, a**	6.0 ± 0.2 **B, a**
1	6.3 ± 0.3 **A, abc**	6.2 ± 0.2 **AB, a**	6.1 ± 0.2 **AB, a**	6.0 ± 0.3 **AB, a**	6.3 ± 0.3 **A, a**	5.9 ± 0.2 **B, a**	5.9 ± 0.4 **AB, a**
2	6.4 ± 0.3 **A, ab**	6.1 ± 0.3 **ABC, a**	6.2 ± 0.1 **AB, a**	6.0 ± 0.3 **BC, a**	6.3 ± 0.2 **AB, a**	5.7 ± 0.2 **C, ab**	6.1 ± 0.2 **ABC, a**
3	6.0 ± 0.2 **A, c**	5.7 ± 0.4 **AB, a**	5.5 ± 0.2 **ABC, b**	5.3 ± 0.4 **BC, b**	5.1 ± 0.3 **C, b**	5.3 ± 0.3 **BC, b**	5.3 ± 0.3 **BC, bc**
4	6.1 ± 0.4 **A, bc**	5.9 ± 0.4 **AB, a**	5.5 ± 0.6 **AB, b**	5.3 ± 0.3 **AB, b**	5.5 ± 0.1 **AB, b**	5.6 ± 0.8 **AB, ab**	5.0 ± 0.2 **B, c**
5	6.3 ± 0.2 **A, abc**	6.1 ± 0.5 **AB, a**	5.4 ± 0.2 **C, b**	5.3 ± 0.3 **C, b**	5.2 ± 0.4 **C, b**	5.3 ± 0.6 **C, b**	5.7 ± 0.3 **BC, ab**

Different capital letters within the same row indicate statistical differences ($P < 0.05$) according to Tukey's HSD

Different lowercase letters within the same column indicate statistical differences ($P < 0.05$) according to Tukey's HSD

Supplementary Table S6: Effect of edible coatings prepared of water, hydro-distilled oregano extract and industrial oregano hydrolate supplemented with 0.5 % oregano essential oil (OEO) on the survival of *S.* Typhimurium FS8 inoculated in pork meat at 4ºC. Inoculated uncoated pork meat samples were used as controls. Sampling was performed immediately after treatment and up to 5 days of storage. Each value represents an average (± standard deviation) of six replicates

				**Extracts**			
**Time (days)**	**Control**	**Water + EO**	**50% Oregano + OEO**	**Centrifuged oregano + OEO**	**50% Centrifuged oregano + OEO**	**Industrial oregano + OEO**	**50% Industrial oregano + OEO**
0	6.5 ± 0.2 **A, a**	5.4 ± 0.2 **B, a**	5.4 ± 0.2 **B, a**	4.8 ± 0.9 **C, a**	5.5 ± 0.4 **B, a**	5.2 ± 0.3 **BC, a**	5.2 ± 0.3 **BC, a**
1	6.3 ± 0.3 **A, abc**	5.2 ± 0.2 **B, abc**	4.8 ± 0.5 **B, bc**	5.2 ± 0.3 **B, a**	5.2 ± 0.4 **B, ab**	4.7 ± 0.3 **B, ab**	4.9 ± 0.2 **B, ab**
2	6.4 ± 0.3 **A, ab**	5.3 ± 0.5 **B, ab**	5.1 ± 0.3 **BC, ab**	4.8 ± 0.3 **BC, a**	5.1 ± 0.2 **BC, ab**	4.7 ± 0.3 **C, ab**	5.0 ± 0.4 **BC, ab**
3	6.0 ± 0.2 **A, c**	4.6 ± 0.3 **BC, c**	4.5 ± 0.3 **BC, c**	4.6 ± 0.7 **BC, a**	4.8 ± 0.1 **B, b**	4.1 ± 0.5 **C, bc**	4.7 ± 0.4 **B, abc**
4	6.1 ± 0.4 **A, bc**	5.1 ± 0.4 **B, abc**	4.3 ± 0.2 **B, c**	4.7 ± 0.4 **B, a**	5.0 ± 0.2 **B, ab**	4.7 ± 0.7 **B, ab**	4.5 ± 0.2 **B, bc**
5	6.3 ± 0.2 **A, abc**	4.8 ± 0.5 **BC, bc**	4.8 ± 0.1 **BC, bc**	4.6 ± 0.5 **BC, a**	5.1 ± 0.2 **B, ab**	3.9 ± 0.4 **D, b**	4.3 ± 0.5 **CD, b**

Different capital letters within the same row indicate statistical differences ($P < 0.05$) according to Tukey's HSD

Different lowercase letters within the same column indicate statistical differences ($P < 0.05$) according to Tukey's HSD
